# Randomized outcome trial of nutrient-enriched formula and neurodevelopment outcome in preterm infants

**DOI:** 10.1186/1471-2431-14-74

**Published:** 2014-03-19

**Authors:** Maria Lorella Giannì, Paola Roggero, Orsola Amato, Odoardo Picciolini, Pasqua Piemontese, Nadia Liotto, Francesca Taroni, Fabio Mosca

**Affiliations:** 1 NICU, Department of Clinical Sciences and Community Health, Fondazione IRCCS Cà Granda Ospedale Maggiore Policlinico, Università degli Studi di Milano, Via Commenda 12, 20122 Milano, Italy

**Keywords:** Preterm infants, Neurodevelopment, Nutrient-enriched formula, Post discharge nutrition

## Abstract

**Background:**

Preterm infants are at risk for adverse neurodevelopment. Furthermore, nutrition may play a key role in supporting neurodevelopment. The aim of this study was to evaluate whether a nutrient-enriched formula fed to preterm infants after hospital discharge could improve their neurodevelopment at 24 months (term-corrected age).

**Methods:**

We conducted an observer-blinded, single-center, randomized controlled trial in infants admitted to the Fondazione IRCCS Cà Granda Ospedale Maggiore Policlinico, University of Milan, Italy between 2009 and 2011. Inclusion criteria were gestational age < 32 weeks and/or birth weight < 1500 g, and being fed human milk for < 20% of the total milk intake. Exclusion criteria were congenital malformations or conditions that could interfere with growth or body composition. Included infants were randomized to receive a standard full-term formula or a nutrient-enriched formula up until 6 months of corrected age, using two computer-generated randomization lists; one appropriate for gestational age (AGA) and one for small for gestational age (SGA) infants. We assessed neurodevelopment at 24 months of corrected age using the Griffiths Mental Development Scale and related subscales (locomotor, personal-social, hearing and speech, hand and eye coordination, and performance).

**Results:**

Of the 207 randomized infants, 181 completed the study. 52 AGA and 35 SGA infants were fed a nutrient-enriched formula, whereas 56 AGA and 38 SGA infants were fed a standard full-term formula. The general quotient at 24 months of corrected age was not significantly different between infants randomized to receive a nutrient-enriched formula compared with a standard term formula up until 6 months of corrected age (AGA infants: 93.8 ± 12.6 vs. 92.4 ± 10.4, respectively; SGA infants: 96.1 ± 9.9 vs. 98.2 ± 9, respectively). The scores of related subscales were also similar among groups.

**Conclusions:**

This study found that feeding preterm infants a nutrient-enriched formula after discharge does not affect neurodevelopment at 24 months of corrected age, in either AGA or SGA infants, free from major comorbidities.

**Trial registration:**

Current Controlled Trials (http://www.controlled-trials.com/ISRCTN30189842) London, UK.

## Background

Preterm infants are at increased risk of adverse neurodevelopmental outcomes [1]. Adequate growth and nutrition may play a key role in facilitating neurodevelopmental performance [2]. Indeed, it is well acknowledged that early nutrition affects brain development both during fetal life and in the first months after birth [3]. In the last decade, attention has focused on nutritional interventions that might improve the growth and development of preterm infants. Tan et al. [4] found that limiting early energy deficits by providing high energy intake in association with increased protein intake during the hospital stay, may support adequate postnatal brain growth and improve neurodevelopmental indices at 3 months of corrected age.

Consistent with these findings, Stephens et al. [5] reported a positive, independent association between first week protein and energy intake, and Mental Developmental Index Scores assessed using the Bayley Scales of Infant Development II, in extremely preterm infants at 18 months of corrected age. To our knowledge, there is paucity of data in the literature concerning the effect of high protein and energy intake after hospital discharge in preterm infants, who are randomized and separately evaluated on neurodevelopmental outcome according to intrauterine growth pattern.

Agosti et al. [6] conducted a multicenter, randomized controlled trial on 121 very low-birth weight infants, randomized at term-corrected age to receive either a preterm formula or a standard term formula up until 55 weeks of corrected age. The authors analyzed the small for gestational age (SGA) infants separately, reporting a higher score on the Griffiths Developmental Scale at 6 months of corrected age in the infants fed with preterm formula compared with the infants fed the standard term formula.

Our group recently conducted a randomized trial that investigated whether consumption of a nutrient-enriched formula after hospital discharge leads to a different growth and weight gain composition in preterm infants, randomized and evaluated according to intrauterine growth pattern.

We tested the hypothesis that preterm infants fed nutrient-enriched formula between term age and 6 months of corrected age would develop lower adiposity compared with preterm infants fed standard formula. Beneficial effects in terms of head circumference growth and fat free mass gain were found in preterm infants born appropriate for gestational age (AGA) who received a nutrient-enriched formula after hospital discharge [7].

In this paper, we report the results concerning the secondary outcome of this randomized controlled study. We evaluated whether the consumption of a nutrient-enriched formula after hospital discharge could affect subsequent neurodevelopment in preterm infants, evaluated separately according to intrauterine growth pattern. We tested the hypothesis that preterm infants who had been fed a nutrient-enriched formula for the first 6 months of corrected age would show higher scores at 24 months of corrected age on the Griffiths Mental Development Scale and related subscales than infants that had been fed a standard formula.

## Methods

### Study design

The study was an observer-blinded, single-center, randomized controlled trial. The results of this study are reported following the CONSORT guidelines. Details of the study are reported elsewhere [6]. The trial was registered with Current Controlled Trials (http://www.controlled-trials.com/ISRCTN30189842) London, United Kingdom.

At term-corrected age, the infants were randomized to receive either a standard full-term formula (treatment A) or a nutrient-enriched formula (treatment B) up until 6 months of corrected age. The introduction of any other food before the infants were 6 months’ corrected age was not allowed. After 6 months, the infants were weaned according to our clinical practice complying with the recommendations of the European Society of Pediatric Gastroenterology and Nutrition [8].

Randomization for feeding with treatment A or B was performed by an independent investigator using two computer-generated randomization lists; one for AGA infants and one for SGA infants, with a random permuted block size of four.

Compared with the standard full-term formula, the nutrient-enriched formula provided higher energy (75 vs. 68 kcal/100 mL), protein (2.0 vs. 1.4 g/100 mL), protein-to-energy ratio (2.6 vs. 2.0 g/100 kcal), carbohydrates (7.5 vs. 7.4 g/100 mL), fat (4.1 vs. 3.7 g/100 mL), docosahexaenoic acid (14 vs. 8.5 mg/100 mL), arachidonic acid (18 vs. 14.9 mg/100 mL), vitamin D (1.7 vs. 1.2 μg/100 mL), calcium (94 vs. 47 mg/100 mL), and phosphorus (50 vs. 33 mg/100 mL). At randomization, the parents were instructed to record the daily quantities of milk consumed by the infants in a diary. The average daily energy (kcal/kg/day) and proteins (g/kg/day) were calculated up until 6 months of corrected age.

#### Subjects

The study was approved by the Ethics Committee of the Fondazione Istituto di Ricovero e Cura a Carattere Scientifico Cà Granda Ospedale Maggiore Policlinico and written informed consent was obtained from the parents.

Among all consecutive newborns admitted to NICU, Fondazione Istituto di Ricovero e Cura a Carattere Scientifico Cà Granda Ospedale Maggiore Policlinico, between 2009 and 2011, 211 preterm infants were enrolled in the study by a single investigator and 194 subjects completed the study at 12 months of corrected age [7]. Inclusion criteria were gestational age < 32 weeks and/or birth weight < 1,500 g and being fed human milk for < 20% of the total milk intake. Exclusion criteria were congenital malformations or conditions that could interfere with growth or body composition (including congenital diseases, chromosomal abnormalities, chronic lung disease, severe brain disease, severe metabolic disease, severe cardiac disease or gastrointestinal diseases).

Neonatal characteristics (gestational age, being AGA or SGA, birth weight, length, and head circumference) were recorded prospectively. Gestational age was based on the mother’s last menstrual period and a first trimester ultrasound examination. Corrected age was calculated using the chronologic age and adjusting for gestational age, i.e., the number of additional weeks from term (40 weeks). Infants were categorized as AGA or SGA if the birth weight was ≥ 10th or < 10th percentile, respectively, according to Fenton’s growth chart [9]. We performed brain magnetic resonance imaging (MRI) after a mean of 40 ± 2 weeks postconceptional age in all newborns. To assess eligibility, brain MRI results were screened for the presence of minor brain lesions (e.g., mild gliosis, mild ventricular dilatation, irregularly shaped ventricles or corpus callosum thinning) [10], as the occurrence of major brain lesions was an exclusion criteria. The maternal educational level (years) was also obtained and categorized as follows: low (≤ 13); high (> 13).

#### Measurements

We assessed anthropometric parameters (weight, length, and head circumference) and neurodevelopment at 24 months of corrected age. We measured anthropometric parameters according to standard procedures [11]. Neurodevelopment was assessed using the Griffiths Mental Development Scale and related subscales (locomotor, personal-social, hearing and speech, hand and eye coordination, and performance) by a single skilled examiner, who was blinded to the infants’ randomization [12]. A sub-quotient for each related subscale was calculated with a mean population value of 100 and a standard deviation of 16. A sub-quotient < 68 was classified as severe developmental delay, whereas a sub-quotient from 68 to 83 indicated mild mental retardation in the domain investigated by the specific subscale.

A general quotient (GQ) was then calculated with a mean population value of 100 and a standard deviation of 12. A GQ < 76 was classified as severe developmental delay. A GQ from 76 to 87 indicated mild mental retardation.

#### Adverse events

Adverse events (AEs) were assessed on the basis of inquires to the parents. All AEs were recorded on adverse event forms and evaluated by the investigator for causality for the relationship to the study feeding and for severity. An AE was defined as any event that was not consistent with the information provided in the consent form or could reasonably be expected to accompany the natural history and progression of the subject’s condition throughout the study. AEs were considered serious if they were fatal or life-threatening, required hospitalization or surgical intervention, resulted in persistent or significant disability/incapacity, or were considered medically relevant by the investigator. All other AEs were categorized as nonserious. AEs were assessed according to body system.

#### Statistical analysis

Sample size was estimated using the value of the GQ. To detect an 11-point (15 SD) difference in the GQ [13] at 5% significance and 80% power, 30 infants in each group were needed. Descriptive data are shown as means ± SD or number of observations (percentages). Comparisons between infants fed treatment A and infants fed treatment B were performed within AGA and SGA groups using the chi square test for discrete variables or analysis of variance for continuous variables. All statistical analyses were conducted at the α = 0.05 level and were two-tailed. Statistical analysis was performed with SPSS 12 (SPSS Inc., Chicago, IL).

## Results

**Figure 1:**
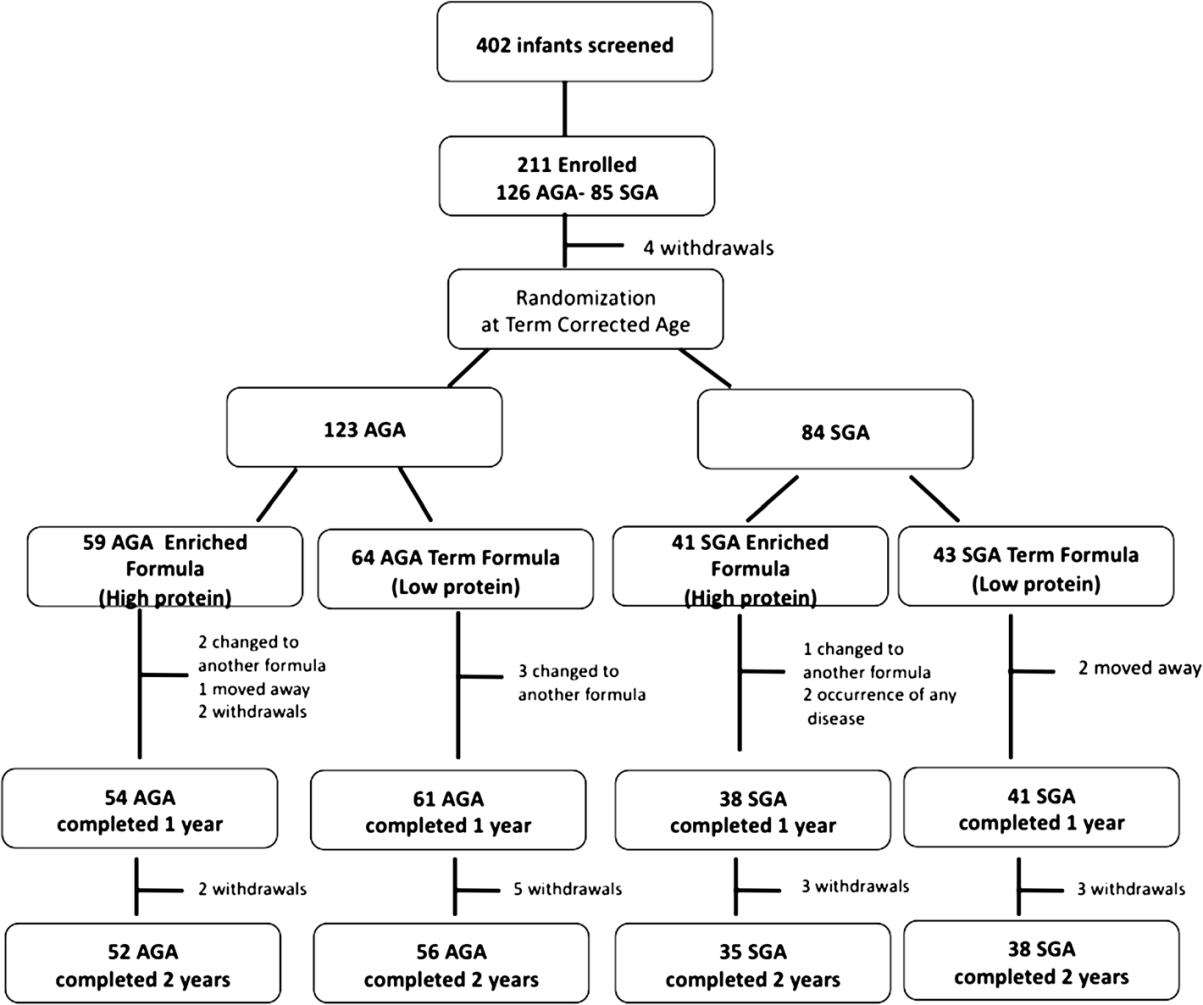
Flow chart of the study.

Of the 194 infants who completed the study at 12 months of corrected age, 181 underwent anthropometric and neurodevelopment assessments (Figure 1). No differences between groups were found in baseline characteristics except for mean protein intake between term and 6 months of corrected age. The latter was higher in infants fed the nutrient-enriched formula than in infants fed the standard term formula, in both AGA and SGA groups (Table 1). Anthropometric characteristics at 24 months of corrected age are presented in Table 2. In Table 3, the infants’ mean GQ and sub-quotients at 24 months are shown. No differences between groups were found either in the GQ or in the sub-quotients.

**Table 1:** Baseline subjects’ characteristics according to randomization

	**AGA**		**SGA**	
	**Treatment B**	**Treatment A**	**Treatment B**	**Treatment A**
	**n = 52**	**n = 56**	**n = 35**	**n = 38**
Gestational age (weeks)	28.9 ± 1.9	29 ± 1.7	31.4 ± 1.9	31.6 ± 1.9
Birth weight (g)	1146 ± 223	1197 ± 204	1103.4 ± 239	1129 ± 304
Birth length (cm)	36.5 ± 3	37.4 ± 2.8	36.5 ± 3.4	36.5 ± 3.8
Birth HC° (cm)	26.4 ± 2	27.1 ± 2.2	27 ± 2.4	27.2 ± 2.4
Twins n (%)	25 (48)	23 (41)	12 (34)	11 (29)
Males n (%)	26 (50)	31 (60)	17 (49)	21 (55)
Abnormal MRI n (%)	5 (10)	2 (4)	4 (11)	5 (13)
Protein intake (g/kg/day)	2.79 ± 0.42*	2.24 ± 0.49	2.94 ± 0.48*	2.26 ± 0.36
(Term-6 months corrected age)				
Energy intake (kcal/kg/day)	109.6 ± 14.3	108.8 ± 17.6	110.2 ± 24.7	108.6 ± 12.7
(Term-6 months corrected age)				
Maternal education >13 years	24 (46)	23 (41)	18 (51)	18 (47)

°HC = head circumference.

*P < 0.001.

Data were analysed using the independent samples T test or the chi square test and comparisons were made between AGA infants fed enriched formula vs AGA infants fed standard formula and between SGA infants fed enriched formula vs SGA infants fed standard formula.

**Table 2:** Growth parameters at 24 months of corrected age according to randomization

	**AGA**		**SGA**	
	**Treatment B**	**Treatment A**	**Treatment B**	**Treatment A**
	**n = 52**	**n = 56**	**n = 35**	**n = 38**
Weight (g)	11930 ± 1637	11662 ± 1219	10682 ± 1249	10793 ± 1445
Length (cm)	86.3 ± 4.3	85.6 ± 2.9	83.1 ± 2.7	83.4 ± 3.1
HC (cm)	48.1 ± 1.6	47.7 ± 1.7	47.7 ± 1.57	47.9 ± 1.17

Data were analysed using the independent samples T test and comparison was made between AGA infants fed enriched formula vs AGA infants fed standard formula and between SGA infants fed enriched formula vs SGA infants fed standard formula.

**Table 3:** Infants’ general quotient and sub-quotients at 24 months according to randomization

	**AGA**		**SGA**	
	**Treatment B**	**Treatment A**	**Treatment B**	**Treatment A**
	**n = 52**	**n = 56**	**N = 35**	**N = 38**
General Quotient	93.8 ± 12.6	92.4 ± 10.4	96.1 ± 9.9	98.2 ± 9
Locomotor	93.7 ± 12.6	95.07 ± 12.7	101.5 ± 6.8	96 ± 13.4
Personal social	88.4 ± 15	86.05 ± 13.7	91.11 ± 13.2	92.7 ± 10.1
Hearing and speech	93.3 ± 13.8	90.9 ± 14	93.6 ± 13.3	96.1 ± 10.2
Hand and eye coordination	97.9 ± 14.6	96.8 ± 10.2	99.7 ± 11.1	103.3 ± 8.09
Performance	96.5 ± 13	96.4 ± 11.3	99.7 ± 7.4	101.3 ± 8.09

Data were analysed using the independent samples T test and comparison was made between AGA infants fed enriched formula vs AGA infants fed standard formula and between SGA infants fed enriched formula vs SGA infants fed standard formula.

### Adverse events

In total, 128 AEs occurred from enrollment up to the end of the study. Of these, 14 occurring in 13 infants were assessed as serious. Documented reasons for all AEs were mostly illnesses that are common during the first two years of life (ie, otitis media, bronchitis, gastroenteritis, upper respiratory tract infection, bronchiolitis, pharyngitis, varicella, urinary tract infection). There were no differences in the incidence of non serious and serious AEs among the groups.

## Discussion

Significant effort has focused on the identification of nutritional approaches applied early in life to improve later neurodevelopment, especially for extremely preterm infants [14].

Contrary to our hypothesis, the present study was unable to show that energy and protein supplementation led to better neurodevelopment of preterm infants at 24 months of corrected age. The absence of a positive effect of nutritional supplementation on neurodevelopment could partially be explained by the fact that the enrolled infants did not develop major comorbidities that could potentially have interfered with neurodevelopment during their hospital stay. However, in our study, the infants’ GQ and sub-quotient scores were normal. This may suggest an overall improvement in care in these vulnerable infants who are at risk for adverse neurodevelopment because brain development occurs primarily from being exposed to non-physiological environmental influences [15].

The absence of any beneficial effect of the nutritional intervention on neurodevelopment at 24 months of corrected age could also be because the post-discharge period may be less sensitive to the influence of energy and protein supplementation than the pre-discharge period [14].

Our findings are consistent with other studies [16-20] that investigated the effect of protein and energy supplementation after discharge on preterm infants’ neurodevelopment. However, it must be underlined that in all but one (ref. [20]) of these studies, the actual energy and protein intake of the enrolled infants was not reported. Therefore, it is not possible to make a comparison with the actual protein and energy intake of infants enrolled in the present study.

Young et al. [16] recently conducted a meta-analysis, including two randomized controlled trials. They concluded that the benefits of using energy and protein supplementation after discharge in preterm infants are not supported by the available evidence.

In one trial [17], randomized 113 preterm infants with a birth weight < 1.75 g and < 34 weeks of gestation, were fed either a preterm infant formula (2.2 g protein/100 ml and 80 kcal/100 ml) or a term formula (1.4 g protein/100 ml and 66 kcal/100 ml) from discharge until 6 months of corrected age. Alternatively, they were fed the preterm formula from discharge to full-term and the full-term formula from full-term to 6 months of corrected age. The authors reported no significant difference in mental or psychomotor development at 18 months of age. In another trial [18], 196 preterm infants weighing < 3.000 g at time of discharge were randomly assigned to receive either a nutrient-enriched post discharge formula (1.85 g protein/100 ml and 72 kcal/100 ml) or a standard full-term formula (1.45 g protein/100 ml and 68 kcal/100 ml) until 9 months’ post term. Developmental scores at 9 or 18 months, assessed using the Bayley Scales of Infant Development II, were similar between the groups. However, infants fed the nutrient-enriched formula had a 2.8 point advantage in the Psychomotor Development Index.

Jeon et al. [19] randomized preterm infants to one of two conditions. Infants received either a preterm formula (2.3 g protein/100 ml and 80 kcal/100 ml) or a term formula (1.6 g protein/100 ml and 67 kcal/100 ml) from term until 6 months of corrected age or a preterm formula to 3 months of corrected age and then term formula to 6 months of corrected age. They reported no significant difference in neurodevelopment outcome at 18 months of corrected age, assessed using the Bayley Scales of Infant Development. Aimone et al. [20] investigated the effect of breast milk fortification after discharge, in a cohort of 39 preterm infants born at < 33 weeks gestation, with birth weights ranging from 750 g to 1800 g. The infants were randomized to receive 12 weeks of either 50% of the daily breast milk intake, fortified to attain a protein and energy content of approximately 2.2 g and 81 kcal/100 ml, or unfortified breast milk. Developmental outcome was assessed at 18 months corrected age, although many subjects were lost to follow-up. The authors reported no significant difference in developmental outcome between groups. Nonetheless, it should be considered that the energy and protein intake at 6 months of corrected age of the infants in both groups of the study by Aimone et al. were much lower than those consumed by the AGA and SGA infants in the present study, irrespective of the type of feeding.

Two trials conducted during a hospital stay have supported the hypothesis that nutritional supplementation improves neurodevelopment [21-24]. However, these studies addressed moderate preterm infants or displayed methodological shortcomings (e.g., small sample size). A beneficial effect of first-week protein and energy intake on medium-term neurodevelopment has been reported in an observational study [5]. However, randomized, controlled trials are superior for cohort analysis because they reduce the effects of known and unknown confounders.

The main strengths of the present study are that the effect of a nutritional intervention on subsequent development was analyzed in subgroups of AGA and SGA preterm infants. Additionally, the actual energy and protein intakes during the intervention period were collected. Indeed, to our knowledge, studies investigating the effect on neurodevelopmental outcome of high protein and energy intake after hospital discharge in preterm infants, who are randomized and evaluated separately according to intrauterine growth pattern, are scarce. Agosti et al. [6] reported interim results from a multi-center, randomized controlled study on 121 very low-birth weight infants. Infants were randomized at term corrected age to receive either a preterm formula (2.4 g/100 ml and 80 kcal/100 ml) or a standard term formula (1.7 g/100 ml and 70 kcal/100 ml) up until 55 weeks of corrected age. The authors focused on infants born SGA, reporting a beneficial effect of the consumption of a preterm formula evidenced by a higher score on the Griffiths Developmental Scale at 6 months of corrected age. However, the authors reported neither the number of infants born SGA that were analyzed nor their actual nutritional intakes.

The main limitation of our study could be that we did not enroll infants affected by major comorbidities. These infants may have benefited most from the nutritional intervention.

## Conclusions

Based on our findings, feeding preterm infants a nutrient-enriched formula after discharge does not appear to affect neurodevelopment at 24 months of corrected age, in either AGA or SGA infants, free form major comorbidities.

Further large, randomized controlled studies are indicated to identify the potential positive effect of energy and protein supplementation on preterm infants when they are older, with a particular focus on the time of school entry.

## Abbreviations

AGA: Adequate for gestational age; SGA: For small for gestational age; MRI: Brain Magnetic Resonance Imaging; GQ: General quotient.

## Competing interests

There authors declare that they have no competing interest.

## Authors’ contributions

MLG: Wrote the paper and contributed to the design of the experiments. PR: Conceived and designed the experiments and contributed to the writing of the manuscript. OA: Analyzed the data. OP: Performed the neurodevelopment assessment. PP: Analyzed the data. NL: Performed the growth assessment. FT: Performed the growth assessment. FM: Provided suggestions with regard to the content and concept of the manuscript. All authors read and approved the final manuscript.

## Pre-publication history

The pre-publication history for this paper can be accessed here:

http://www.biomedcentral.com/1471-2431/14/74/prepub
